# The lactate receptor HCAR1: A key modulator of epileptic seizure activity

**DOI:** 10.1016/j.isci.2024.109679

**Published:** 2024-04-06

**Authors:** Maxime Alessandri, Alejandro Osorio-Forero, Anita Lüthi, Jean-Yves Chatton

**Affiliations:** 1 Department of Fundamental Neurosciences, University of Lausanne, 1005 Lausanne, Vaud, Switzerland

**Keywords:** Molecular biology, Neuroscience

## Abstract

Epilepsy affects millions globally with a significant portion exhibiting pharmacoresistance. Abnormal neuronal activity elevates brain lactate levels, which prompted the exploration of its receptor, the hydroxycarboxylic acid receptor 1 (HCAR1) known to downmodulate neuronal activity in physiological conditions. This study revealed that HCAR1-deficient mice (HCAR1-KO) exhibited lowered seizure thresholds, and increased severity and duration compared to wild-type mice. Hippocampal and whole-brain electrographic seizure analyses revealed increased seizure severity in HCAR1-KO mice, supported by time-frequency analysis. The absence of HCAR1 led to uncontrolled inter-ictal activity in acute hippocampal slices, replicated by lactate dehydrogenase A inhibition indicating that the activation of HCAR1 is closely associated with glycolytic output. However, synthetic HCAR1 agonist administration in an *in vivo* epilepsy model did not modulate seizures, likely due to endogenous lactate competition. These findings underscore the crucial roles of lactate and HCAR1 in regulating circuit excitability to prevent unregulated neuronal activity and terminate epileptic events.

## Highlights

• The lactate receptor HCAR1 is expressed by neurons of the CNS in rodents and humans
• HCAR1 plays an important role in mitigating epileptic seizures in mice
• Lack of HCAR1 is associated with increased seizure severity, duration, and mortality
• HCAR1 activation is tightly linked to tissue glycolytic output during seizures

## Graphical abstract

**Figure:**
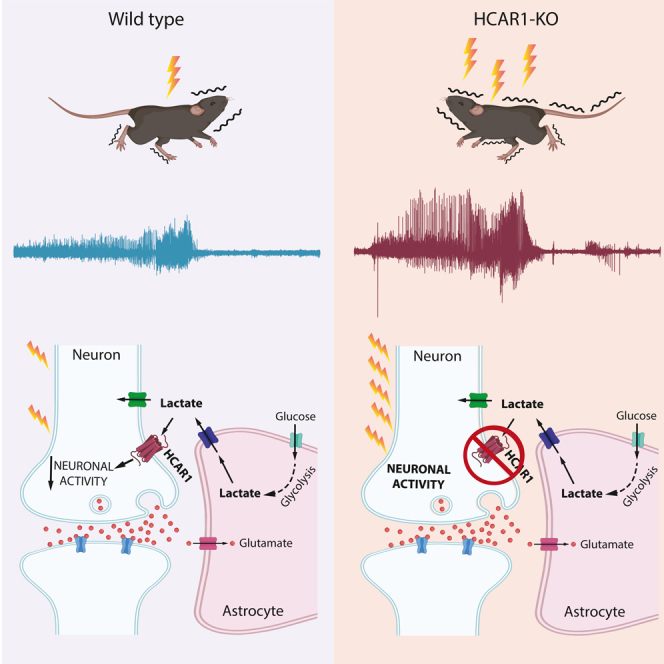

## Introduction

Epilepsy affects about 1% of the world population. Given the current worldwide population, about 80 million individuals suffer from this chronic neurological disorder. It is marked by recurrent seizures and significantly impacts patients' quality of life. It is characterized by abnormal brain activity, leading to diverse manifestations such as generalized convulsions.^1^

Numerous studies have demonstrated that brain lactate levels can exceed 6mM in the extracellular space during seizures,^2^^,^^3^^,^^4^^,^^5^^,^^6^ a concentration sufficient to activate the lactate receptor, known as the hydroxycarboxylic acid receptor 1 (HCAR1).^7^^,^^8^ Given the expression of HCAR1 mainly associated with excitatory neurons of the hippocampus,^9^ a known seizure initiation site,^10^^,^^11^^,^^12^^,^^13^ the ability of the lactate receptor to dampen neuronal excitability makes it particularly significant in the context of epilepsy and places it as a promising target for developing novel antiepileptic drugs.^6^^,^^14^ Furthermore, HCAR1 presence and down-modulating effect on neurons were found in mouse, rat, and human brains.^9^

Lactate has long been considered a waste product of metabolism. Its presence in tissues was long viewed as a hallmark of incomplete respiration and lack of oxygen. It is only in recent years that lactate emerged as a pivotal molecule for numerous processes, including within the central nervous system. Neurons can use lactate as an energy substrate during periods of increased activity, a phenomenon known as the astrocyte-neuron lactate shuttle (ANLS).^15^^,^^16^^,^^17^ This mechanism involves astrocytes detecting increased neuronal activity and responding by releasing lactate subsequently used as a metabolic substrate by neurons.

The role of lactate has expanded beyond being an energy substrate. It has now become evident that lactate also serves as a signaling molecule (for comprehensive reviews see Brooks, 2020; Mosienko et al., 2015).^18^^,^^19^ Previous studies have identified synthetic non-metabolized agonists, such as 3Cl-HBA, that can activate HCAR1 with higher affinity than lactate.^20^ The activation of the receptor initiates an intracellular signaling cascade that reduces spontaneous neuronal firing and excitability.^7^^,^^8^^,^^9^^,^^21^^,^^22^^,^^23^ The molecular mechanisms following HCAR1 activation involve the cyclic AMP-protein kinase A pathway.^8^^,^^24^ In the hippocampus, we and others described that its activation leads to a decrease in neuronal firing and excitatory postsynaptic currents.^9^^,^^23^

Our objective was to delineate the role of HCAR1 in seizure modulation using both *ex vivo* and *in vivo* approaches, and by applying a combination of electrophysiological recordings and behavioral assessments. We propose that lactate exerts a tonic inhibition during epileptic seizures through its activation of HCAR1.

## Results

### Hydroxycarboxylic acid receptor 1 increases seizure threshold and survival

**Figure 1:**
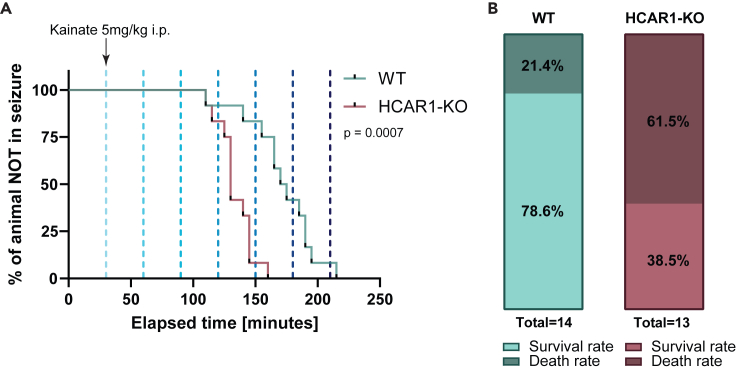
Increased sensitivity of HCAR1-KO mice to kainate-induced seizures (A) Kaplan-Meier plot illustrating the time required for a mouse to develop a clinical seizure of score 4 on the Racine scale following consecutive kainate injections (5 mg/kg, i.p.) at 30-min intervals. *p* = 0.0007, Gehan-Breslow-Wilcoxon test, *n* = 12 animals per group. (B) Comparison of death rates between WT and HCAR1-KO groups after acute seizures induced by two consecutive subcutaneous injections of kainate administered 1 h apart.

Kainic acid is one of the most widely used chemical agents to induce seizures in rats and mice. Its injection, whether intracerebral or systemic, leads to the development of limbic seizures originating in the hippocampus and closely resembling the features found in humans.^25^ To investigate the propensity of HCAR1-KO mice to develop seizures, repeated injections of subthreshold doses of kainate (5 mg/kg, i.p.) were administered at 30-min intervals.^26^ Using this model, we observed that HCAR1-KO mice display a lower seizure threshold, as evidenced by a leftward shift in the HCAR1-KO survival curve (Figure 1A). In addition, HCAR1-KO mice were more likely to die (61.5%) during seizures induced by subcutaneous injections of kainate (Figure 1B) compared to the WT (21.4%). These results highlight that HCAR1-KO mice have an increased susceptibility to seizures and are at a higher risk of death during seizures, suggesting that HCAR1 might have a protective effect.

### Hydroxycarboxylic acid receptor 1 reduces the number of seizures and their duration

**Figure 2:**
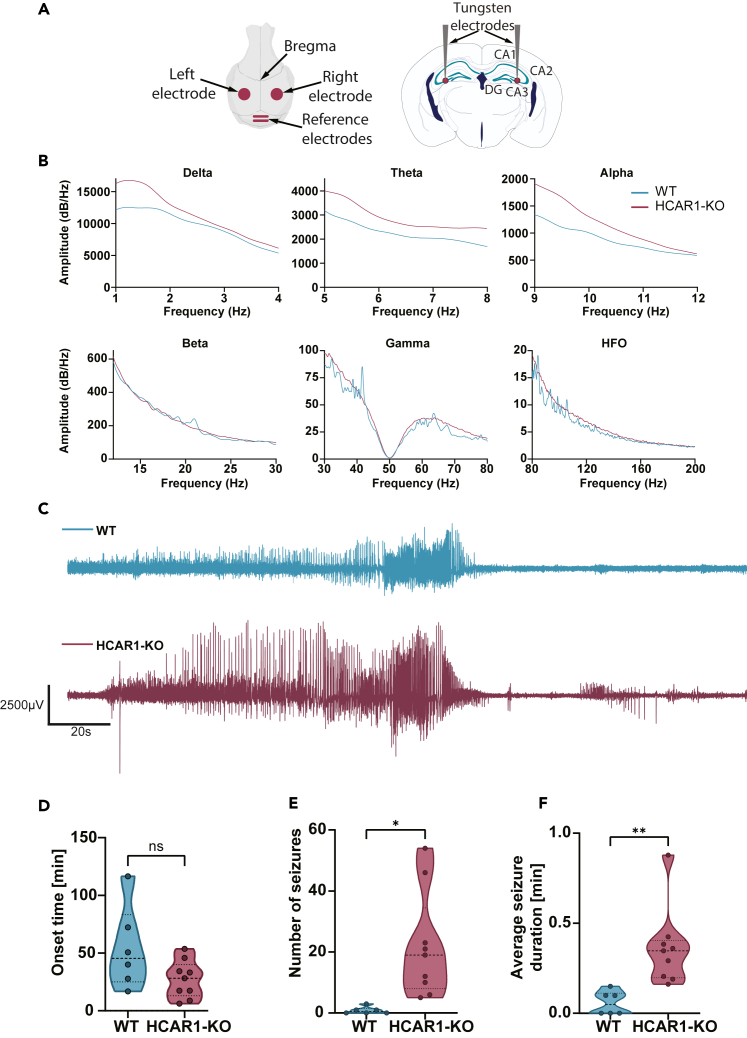
Increased seizure number and duration in HCAR1-KO mice (A) Schematic depiction of LFP electrode placement in the dorsal CA3 region of the hippocampus, presented in top (left) and coronal (right) views. (B) Mean frequency power in baseline condition (20 min of wakefulness) in WT and HCAR1-KO mice. HCAR1-KO mice have increased baseline power in the low-frequency range compared to WT. In the gamma panel, the drop at 50Hz is the result of the notch filtering. (C) Representative LFP traces showing an ictal event in a WT and HCAR1-KO mouse. Traces show an overall higher and longer activity in the HCAR1-KO. (D) Onset time from kainate injection to first detected seizure. Means ± SEM: WT = 54.04 ± 14.75, HCAR1-KO = 27.23 ± 5.4 *p* = 0.07 *n* = 6 WT, *n* = 9 HCAR1-KO *p* = 0.07. (E) Number of detected seizures after two subcutaneous injections of kainate (10 mg/kg) recorded for 3 h. Means ± SEM: WT = 0.83 ± 0.5, HCAR1-KO = 21.78 ± 5.8. *p* = 0.0119 unpaired t-test, *n* = 6 WT, *n* = 9 HCAR1-KO. (F) Mean seizure duration in minutes in the WT and HCAR1-KO after two subcutaneous injections of kainate (10 mg/kg). Means ± SEM: WT = 0.057 ± 0.03, HCAR1-KO = 0.36 ± 0.07. *p* = 0.0058 unpaired t-test, *n* = 6 WT, *n* = 9 HCAR1-KO.

To quantify the number of seizures in the hippocampus, tungsten electrodes were implanted, one each in the two CA3 regions of the dorsal hippocampus (Figure 2A). A 1-h baseline recording was taken and used to detect seizure and normalize frequencies in decibel scale. From this baseline, a 20-min recording of wakefulness was used to assess the frequency spectrum in WT and HCAR1-KO mice (Figure 2B). HCAR1-KO displayed increased frequency power in the delta, theta, and alpha range but not in beta, gamma, and HFO. Acute seizures were induced by two injections of kainate (10 mg/kg s.c) at 1-h intervals. Representative traces showing an ictal event in both genotypes are shown in Figure 2C. HCAR1-KO mice displayed a tendency to develop seizure faster (*p* = 0.07) compared to the WT (Figure 2D). HCAR1-KO mice showed a significant increase in the number of detected seizures compared to the WT (Figure 2E). Moreover, 50% (3 out of 6) of the WT failed to develop any seizures, a phenomenon not observed in the HCAR1-KO mice. Following the same trend, HCAR1-KO mice developed significantly longer seizures compared to the WT (Figure 2F). These data, in line with the previous results, suggest that HCAR1-KO mice experience more frequent and longer seizures, which might indicate that HCAR1 could exert a tonic inhibition during seizures. These results prompted us to quantify seizure severity in the frequency domain of the EEG signal.

### Hydroxycarboxylic acid receptor 1 participates in reducing frequency power in mice electroencephalogram

**Figure 3:**
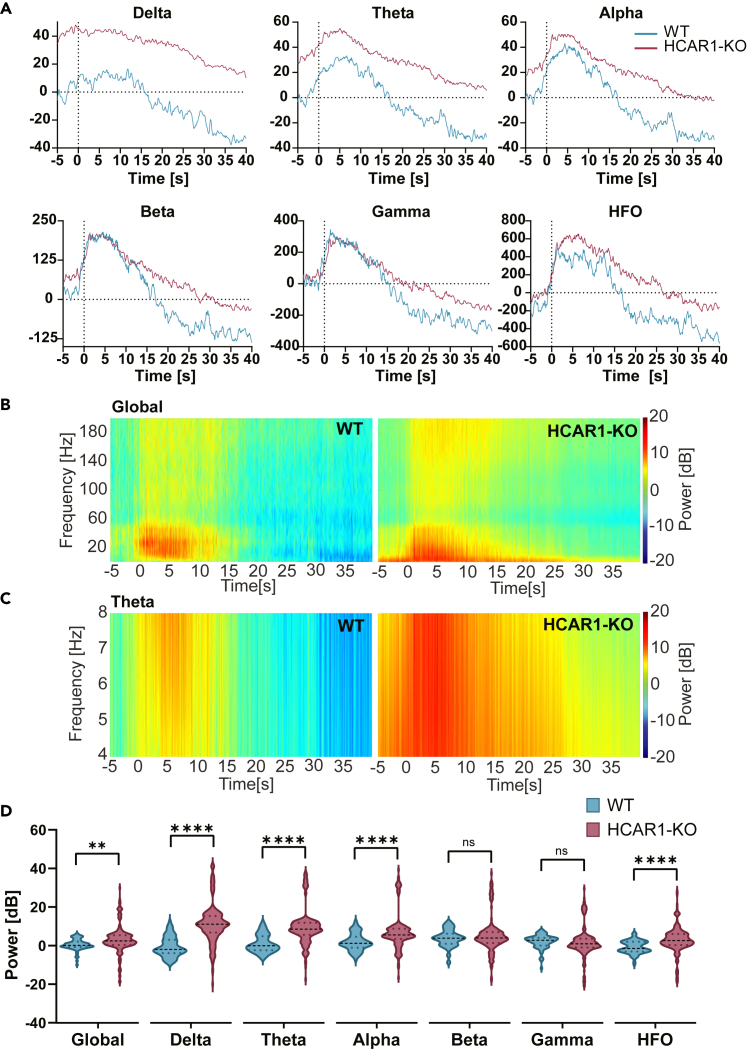
EEG frequency band quantification during epileptic seizures (A) Frequency bands dynamic during epileptic seizures in WT and HCAR1-KO mice. HCAR1-KO mice require more time to return to baseline levels and display increased power in the low frequencies. (B and C) Spectrograms depicting the average seizure activity in WT and HCAR1-KO mice for the Global and Theta frequency bands. Pseudocolors represent the power in dB of each frequency at each time point. Color scales are shown next to the graphs. *n* = 72 seizures over 8 animals for WT and *n* = 196 seizures over 9 animals for HCAR1-KO. (D) Power in dB for the frequency bands during epileptic seizures in WT and HCAR1-KO mice, averaged over time following two subcutaneous injections of kainate. *Global (0-200Hz)*: Means ± SEM: WT = 0.44 ± 0.34, HCAR1-KO = 3.22 ± 0.49, *p* = 0.0012. *Delta (1-3Hz)*: Means ± SEM: WT = −0.62 ± 0.60, HCAR1-KO = 11.32 ± 0.72, *p* < 0.0001. *Theta (4-8Hz)*: Means ± SEM: WT = 0.99 ± 0.53, HCAR1-KO = 9.47 ± 0.66, *p* < 0.0001. *Alpha (9-13Hz)*: Means ± SEM: WT = 1.82 ± 0.51, HCAR1-KO = 7.00 ± 0.66, *p* < 0.0001. *Beta (14-30Hz)*: Means ± SEM: WT = 3.83 ± 0.52, HCAR1-KO = 5.22 ± 0.63, *p* = 0.211. *Gamma (31-80Hz)*: Means ± SEM: WT = 2.06 ± 00.42, HCAR1-KO = 1.78 ± 0.55, *p* = 0.77. *HFO (80-200Hz)*: Means ± SEM: WT = −0.75 ± 0.38, HCAR1-KO = 2.93 ± 0.47, *p* < 0.0001. Unpaired t-test, *n* = 72 seizures over 8 animals for WT and *n* = 196 seizures over 9 animals for HCAR1-KO.

Due to the lower sensitivity to kainate-induced seizures in the WT, it became necessary to increase the kainate dosage for this group to reliably induce seizures in a sufficient number of mice. Therefore, for the remainder of this study, WT mice were injected with 2 × 15 mg/kg s.c. instead of 2 × 10 mg/kg. The dosage for the HCAR1-KO mice remained unchanged for ethical reasons, as these mice already exhibited a significant mortality rate at these kainate doses. Following the same procedure as before, tungsten electrodes were implanted in the hippocampus, and acute seizures were induced by 2 subcutaneous injections of kainate at 1-h intervals. Quantification of seizure power through complex Morlet wavelets convolution was performed with the major frequency bands. HCAR1-KO mice showed increased frequency power (Figure 3A) in delta, theta, and alpha range. This increase is homogeneous from the seizure onset to the end of the analysis window. HCAR1-KO mice also do not return to baseline level or take longer to do so. In higher frequencies, the increased power is not present at seizure onset. However, HCAR1-KO mice display the same increased recovery time as for lower frequencies. This increase in frequency power can be appreciated in the spectrograms in Figures 3B and 3C. Quantification of seizure power averaged over time showed a significant increase in frequency range (global 0-200Hz), in delta (1-3Hz), theta (4-8Hz), alpha (9-13Hz), and HFO (81-200Hz; Figure 3D). These results further underscore the critical role of HCAR1 in the regulation of pathological convulsive activity.

### Hydroxycarboxylic acid receptor 1 broadly impacts seizures across the brain

**Figure 4:**
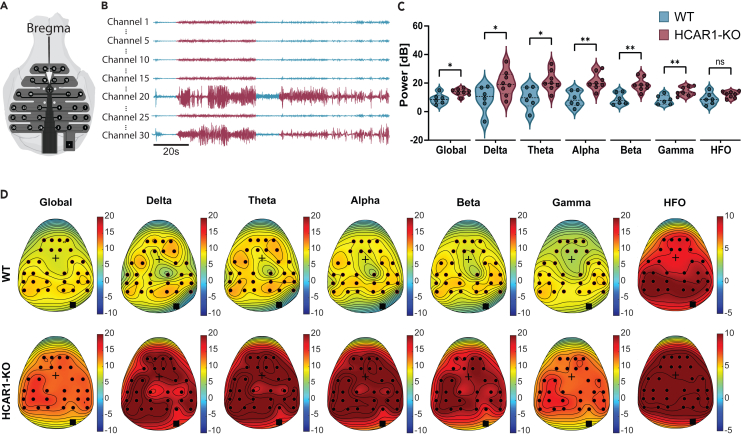
Spatial distribution of EEG power during seizures (A) Schematic representation of the EEG electrode array implant comprising 30 electrodes. (B) Example traces of 7 out of 30 channels. Detected seizure activity is highlighted in red in the traces. (C) Power of the different frequency bands for the average seizure in the WT and HCAR1-KO averaged over time and channels. *Global (0-200Hz)*: Means ± SEM: WT = 8.86 ± 1.47, HCAR1-KO = 13.67 ± 0.84, *p* = 0.0110. *Delta (1-3Hz)*: Means ± SEM: WT = 9.09 ± 3.70, HCAR1-KO = 19.85 ± 3.14, *p* = 0.0046. *Theta (4-8Hz)*: Means ± SEM: WT 9.540 ± 3.077, HCAR1-KO 21.44 ± 2.48, *p* = 0.0103. *Alpha (9-13Hz)*: Means ± SEM: WT = 8.88 ± 2.218, HCAR1-KO = 21.20 ± 2.104, *p* = 0.0019. *Beta (14-30Hz)*: Means ± SEM: WT = 9.005 ± 1.69, HCAR1-KO = 19.09 ± 1.62, *p* = 0.0012. *Gamma (31-80Hz)*: Means ± SEM: WT = 8.253 ± 1.313, HCAR1-KO = 14.12 ± 1.08 *p* = 0.004. *HFO (80-200Hz)*: Means ± SEM: WT = 9.07 ± 1.70, HCAR1-KO = 11.95 ± 0.80. *p* = 0.1228, unpaired t test, *n* = 6 WT, *n* = 8 HCAR1-KO. (D) Topographical maps of the average seizure in WT and HCAR1-KO mice showing the spread of the power through the brain according to frequency bands. Pseudocolors represent the power in dB of the frequency bands at each point in space. *n* = 6 WT, *n* = 8 HCAR1-KO.

In the hippocampus, our research has shown that the HCAR1 primary influence is to downregulate neuronal activity.^9^ However, it remains to be seen whether this effect is consistent throughout the entire brain. To address this question, we employed a high-density EEG electrode array placed on the surface of the skull (Figure 4A) to simultaneously record seizures across 30 channels (Figure 4B). The quantification of seizure power, averaged over channels and time, was consistent with the measurement done in the hippocampus, revealing that HCAR1-KO mice exhibit enhanced power across most frequency bands (Figure 4C). Topographical maps representing the averaged power over time indicate that this effect is not spatially constraint but appears to impact the entire brain uniformly (Figure 4D). Individually, most of the recorded mice showed uniform EEG power across the brain. A few mice, however displayed locally identifiable foci with higher or lower EEG power. However, their localizations were variable, and no distinct patterns could be extracted (Figures S1 and S2).

### Hydroxycarboxylic acid receptor 1 agonist fails to modulate seizure *in vivo*

**Figure 5:**
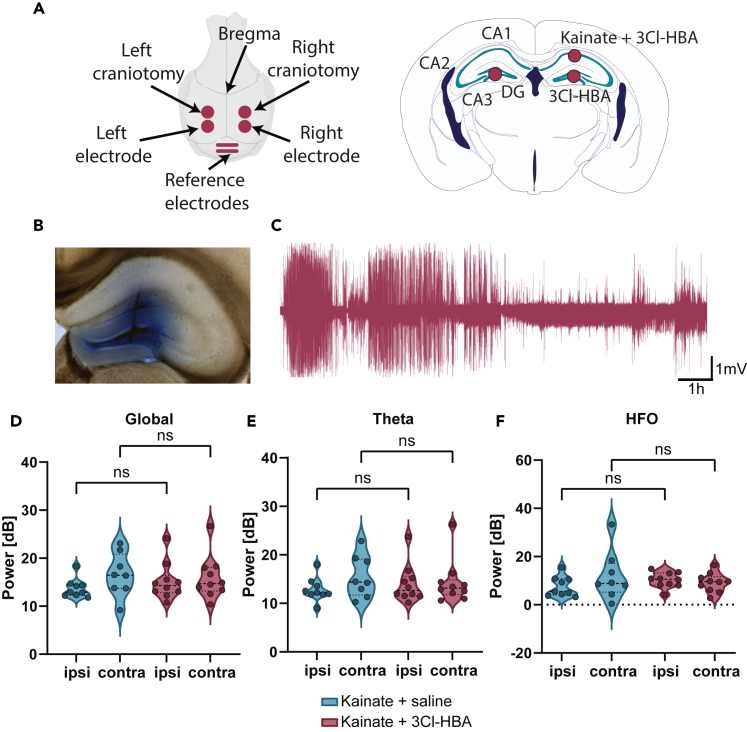
HCAR1 agonists fail to modulate seizures in the intrahippocampal kainate injection model (A) Schematic representation of subdural electrode placement over the dorsal hippocampus and the craniotomies for kainate injection. Left: top view. Right: Coronal view depicting the injection points as red circles. (B) Image of the 3Cl-HBA injection point in the dentate gyrus after the injection of Evan’s blue dye. (C) Example trace of an EEG recording displaying typical seizure events. (D–F) Power of the frequencies in the ipsilateral and contralateral hemispheres during seizures for (D) Global (0-500Hz): Means ± SEM: saline ipsilateral = 13.74 ± 0.67, saline contralateral = 16.58 ± 1.59, 3Cl-HBA ipsilateral = 15.13 ± 1.21, 3Cl-HBA contralateral = 15.93 ± 1.42. *p* > 0.005. (E) Theta (4-8Hz): Means ± SEM: saline ipsilateral = 12.83 ± 0.81, saline contralateral = 15.50 ± 1.53, 3Cl-HBA ipsilateral = 14.05 ± 1.24, 3Cl-HBA contralateral = 14.30 ± 1.42. *p* > 0.005. (F) HFO (80-500Hz): Means ± SEM: saline ipsilateral = 7.53 ± 1.40, saline contralateral = 11.94 ± 3.59, 3Cl-HBA ipsilateral = 10.25 ± 0.99, 3Cl-HBA contralateral = 9.17 ± 1.24. *p* > 0.005. Two-way ANOVA *n* = 9 control, *n* = 10 3Cl-HBA (2.5mM).

Given the increased seizure severity found in HCAR1-KO mice in comparison to their WT counterpart, we attempted to mitigate seizure severity by utilizing the synthetic HCAR1 agonist, 3Cl-HBA.^20^ In the absence of available pharmacokinetic data for 3Cl-HBA, we administered the drug directly into the hippocampus and recorded seizures using subdural electrodes (Figures 5A and 5C). The agonist was injected into the dentate gyrus (Figure 5B) due to the notably high expression of HCAR1 in hilar mossy cells^9^ which have been found to play a significant role in seizure initiation.^11^ According to the dye diffusion assay (Figure 5B), the drug is expected to spread more globally within the hippocampus and the kainate injection in the CA1 was supplemented with the agonist. With this combination of factors, we expected that the agonist would affect a broad area within the hippocampus. Seizure power did not exhibit significant differences between the two conditions (Figures 5D–5F), as shown for the global, theta, and HFO frequencies, that correspond to the most relevant bands generated within the hippocampus and during seizures.^27^^,^^28^ The analysis of other frequency bands (not shown) did not show significant differences. Furthermore, we quantified additional EEG metrics (not shown), such as standard deviation, zero crossing rate, root-mean-square , Kurtosis, skewness, spectral entropy, and power spectral density, none of which displayed significant differences. As a result, we concluded that the EEG signals recorded during seizures remained remarkably similar, whether 3Cl-HBA was administered or not.

### Hydroxycarboxylic acid receptor 1 is endogenously activated during seizures *ex vivo*

**Figure 6:**
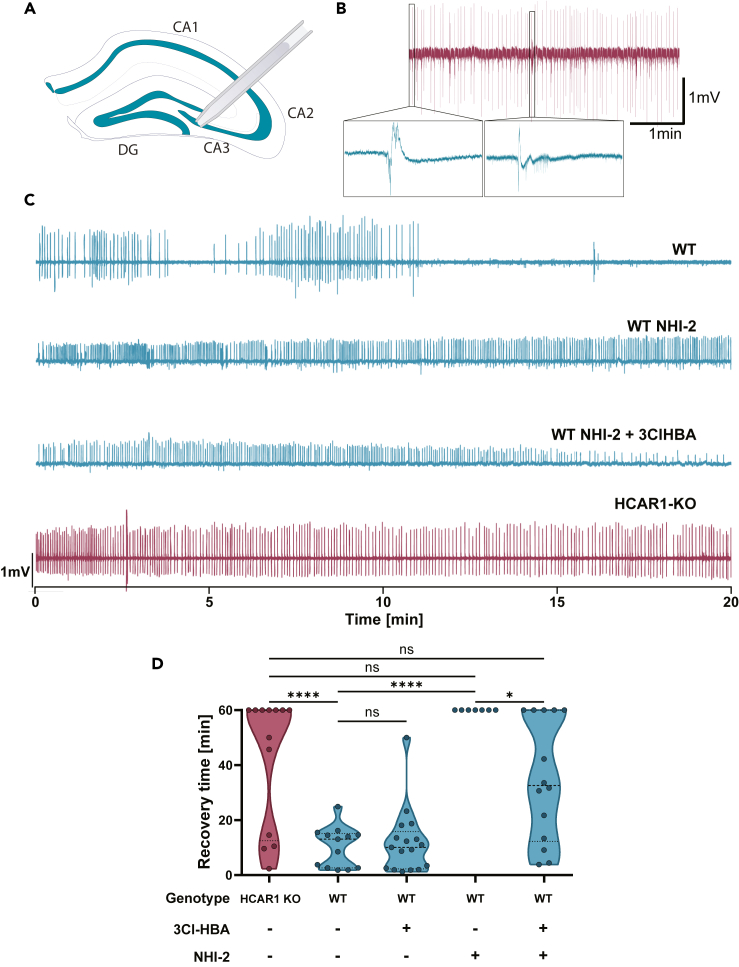
Glycolysis-dependent activation of HCAR1 during seizure *in vitro* (A) Schematic representation of the glass electrode placement in the CA3 in acute hippocampal slices. (B) Example trace of a recording displaying seizure-like events with two zoomed-in interical events (inserts). (C) Representative traces of a 20-min recording after the perfusion of normal aCSF in the WT, WT with 6μM of NHI-2, WT with 6μM of NHI-2 and 80μM of 3Cl-HBA, and HCAR1-KO mice. (D) Quantification of the recovery time after washout of the epileptic aCSF in WT mice in the presence or absence of HCAR1 agonist in control or under the inhibition of LDHA. Means ± SEM: control = 10.31 ± 2.02 *n* = 13, 3Cl-HBA (80μm) = 11.82 ± 2.88 *n* = 17, NHI-2 (6μm) = 60.00 *n* = 7, NHI-2 (6μm) + 3Cl-HBA (80μm) = 35.05 ± 5.94 *n* = 14. *p* = 0.0001 (HCAR1-KO vs WT control), *p* < 0.001 (WT control vs NHI-2), *p* = 0.018 (NHI-2 vs NHI-2 + 3Cl-HBA). Ordinary one-way ANOVA with Tukey’s multiple comparisons test. Recordings were stopped after 60 min.

It is well-documented that during seizures, lactate levels can surge to more than six times their resting concentration^2^^,^^3^^,^^4^^,^^5^ and according to the ANLS hypothesis, astrocyte lactate release is regulated by neuronal activity. We therefore hypothesized that HCAR1 might be activated by endogenous lactate during the increased activity found in seizures. To investigate this hypothesis, we moved to an *in vitro* model of epilepsy. We induced seizures in hippocampal slices using 100μM 4-AP and 0Mg^2+^ and recorded interictal-like activity in the CA3 region (Figures 6A–6C), a well-known seizure initiation site *in vitro.*^10^

The time to seizure onset, i.e., the time for the abnormal activity to start after the perfusion of epileptic artificial cerebrospinal fluid (aCSF), did not exhibit significant differences between HCAR1-KO mice and the WT (Figure S3A). Consistent with what has been observed *in vivo*, the activation of HCAR1 did not affect seizure onset (Figure S3B). We then quantified the recovery time after returning to a non-epileptic aCSF. We discovered that the absence of HCAR1 markedly prolonged the time required for abnormal activity to cease (Figure 6D) to such an extent that 7 out of 13 slices still displayed epileptic-like events after 1h of recording in normal aCSF. Following this finding, we focused our attention on the recovery time. In line with previous data (Figures 5D–5F), the activation of HCAR1 with 3Cl-HBA did not significantly impact recovery time (Figure 6D). To test our initial hypothesis, we prevented the glycolytic synthesis of lactate in astrocytes by selectively blocking the LDHA.^29^ Under these circumstances, the recovery time in the WT was mimicking the one found in HCAR1-KO mice (Figures 6D and S3C) consistent with the hypothesis that lactate levels were sufficient to bind to HCAR1. Under these conditions, the activation of HCAR1 by 3Cl-HBA reduced the recovery time (Figure 6D). These results suggest that HCAR1 is endogenously activated during seizures.

## Discussion

Lactate is no longer to be considered a toxic waste product of metabolism. It has evolved to a multifaceted signaling molecule and mounting evidence have shown that it is able to induce several signaling cascades over the whole body.^19^ Specifically in the brain, lactate exerts significant effects with a variety of responses.^30^^,^^31^^,^^32^^,^^33^ Lactate has been found to have neuroprotective properties in pathologies such as ischemia.^34^ Lactate is known to modulate cellular redox state^35^ and was recently shown to increase two-pore-domain potassium channel activity and expression on astrocytes,^36^ providing yet another potential protective role against excitotoxicity.

The discovery of the lactate receptor has added a new layer of complexity to our view of lactate. Indeed, some effects of lactate have been found to be receptor-mediated,^7^^,^^8^^,^^32^ while others seem to have a different, probably metabolic, origin.^34^^,^^37^ The neuroprotective roles of HCAR1 are still unclear,^37^ however, recent studies suggest that HCAR1 may display protective properties in neonatal stroke^38^ and could promote tissue repair and neurogenesis in the hippocampus.^39^ In the present study we are providing evidence that lactate, through its action on HCAR1, serves as a guardian of circuit homeostasis to prevent a runaway of neuronal activity.

Recently, several key metabolic pathways have been investigated as potential targets to treat epilepsy,^40^ with glycogen and glucose metabolism emerging as important components. Metabolic therapies focus on reducing the amount of energy available to neurons typically by inhibiting metabolic pathways^41^^,^^42^ or modulating metabolite transport.^43^ This approach might be challenged by the observation that 2-deoxy-D-glucose could facilitates seizure-like activity via an excitatory mechanism dependent on oxidative phosphorylation impairment.^44^ The ketogenic diet is the prime example that modifying metabolism is an effective way of epilepsy management. Interestingly, lactate has been found to be elevated under ketogenic treatment in the cerebrospinal fluid of epileptic patient suffering from GLUT1 deficiency syndrome.^45^

Here, we present a novel mechanism through which lactate contributes to regulating the network, thereby expanding the multifaceted roles of lactate within the brain. The ANLS suggests that, as neural activity increases, lactate availability also rises which can fuel the neurons to sustain firing. Such a mechanism could lead to a feedforward loop promoting increased neuronal firing. HCAR1 exhibits a relatively low affinity for lactate. The EC_50_ of HCAR1 for lactate was measured in primary neuronal culture at 4.2mM.^7^ Similar values were found in other cells.^46^ It means that HCAR1 effects come into play when lactate reaches significant levels, such as the 6mM found during seizure in humans.^2^^,^^3^^,^^4^^,^^5^ Accordingly, HCAR1 potentially serves as a warning signal for excessive neural activity. This hypothesis is supported by the decreased seizure threshold that we observed in mice lacking HCAR1. The active brain is governed by a fine balance between excitation and inhibition. In the absence of HCAR1, this balance is disrupted already during baseline recordings where HCAR1-KO mice demonstrated increased low-frequency power, a hallmark of synchronization. Moreover, during seizure activity HCAR1-KO mice have an even higher increase in frequency power. The decreased threshold in HCAR1-KO mice as well as the increased frequency of seizures, suggests that HCAR1 plays an important homeostatic role for delaying seizure onset.

Often overlooked, seizure termination is a critical factor, as failure to halt seizures can lead to status epilepticus—a life-threatening condition associated with poor outcomes and numerous sequelae.^47^ It is defined as long-lasting seizures (more than 30 min) or as several consecutive seizures without regaining consciousness^48^ that arises when natural mechanisms that terminate seizures fail. Our findings introduce the possibility that lactate, previously regarded as detrimental for epilepsy,^42^^,^^49^ may function as an essential endogenous neuromodulator, actively contributing to seizure termination both *in vivo* and *ex vivo,* much like adenosine,^50^ endocannabinoids,^51^ or neuropeptide Y.^52^

The broad impact of HCAR1 became evident when considering that its absence resulted in increased seizure severity across various EEG frequency bands, spanning not only the hippocampus but the entire brain. This observation underscores the extensive reach of lactate in the brain.

The theta rhythm is one of the strongest extracellular oscillations of the brain^53^ and a hallmark of hippocampal circuitry. Mainly controlled by extrahippocampal structure such as the medial septum^54^ it has been shown to also arise from intrinsic properties of pyramidal neurons both in the CA1^55^ and in the CA3.^56^ It has also been found to be generated by hilar mossy cells in the dentate gyrus.^11^^,^^27^ These three cell types have been found to express HCAR1 at various levels.^9^ The disrupted theta rhythm that we observed in HCAR1-KO mice, although artificially induced by kainate in this study, suggests potential repercussions under physiological conditions. Given the established role of theta rhythm in cognitive processes and behavior,^57^ this disturbance may contribute to the differential impact of lactate on memory consolidation attributed to HCAR1 in previous studies.^58^

High-frequency oscillations (HFOs) are established hallmarks of epilepsy and serve as biomarkers of epileptogenesis.^59^ Their spatial localization is used by neurosurgeons during presurgical examination for defining the seizure onset zone and therefore, the area to resect in patients with pharmacoresistant epilepsy.^60^ While the exact mechanisms underlying HFO generation remain incompletely understood, studies suggest that HFOs below 250Hz result from fast-spiking neurons in the dentate gyrus.^61^^,^^62^ Intriguingly, these oscillations specifically originate in the hilar region of the dentate gyrus and are not dependent on gap junctions,^61^ ruling out the implications of electrical synapses of interneurons which suggest a role for hilar mossy cells.

Hilar mossy cells, often overlooked due to their substantial loss in mesial temporal lobe epilepsy,^63^ have gained attention within the last 5 years as potential drivers of epileptic seizures.^11^^,^^12^^,^^64^ We have confirmed the abundant expression of HCAR1 in mossy cells and demonstrated its ability to attenuate their activity on acute slices.^9^ Our findings of high HFO rates in HCAR1-KO mice suggest that HCAR1 can modulate these cells, potentially linking them and HCAR1 to epileptogenesis. Taken together, our results underscore the importance of investigating HCAR1 as a potential avenue for new therapeutic approaches in epilepsy.

Currently available HCAR1 agonists have a low affinity for HCAR1^8^^,^^20^^,^^65^ and in addition, the presence of high interstitial lactate concentration during ictal activity likely hinders the potential effects of exogenous agonists. Their use for treatment may therefore not be the most suitable approach. A more promising strategy may involve the use of GPCR allosteric modulators to enhance the effect of the endogenous lactate on HCAR1.^66^^,^^67^ While such modulators have not yet been identified for HCAR1, it is worth noting that many other GPCRs, including the adenosine receptor A1 and GABA_B_ receptor, are sensitive to allosteric modulation.^66^ This would allow the activation of HCAR1 preemptively before the surge of lactate, potentially mitigating seizure onset. Moreover, we previously established that HCAR1 expression and function are not limited to mice but appear to be conserved in rat and human brain tissue.^9^ This suggests that HCAR1 may have an interesting translational potential when considered for therapies.

Collectively, our experiments conducted on acute seizure models along with the analysis of brain activity, would support the notion that in chronic epilepsy, a significantly higher proportion of mice lacking HCAR1 would exhibit spontaneous recurrent seizures in comparison to WT mice. Accordingly, it appears reasonable to postulate that mice lacking HCAR1 would encounter more pronounced, longer, and more frequent episodes of spontaneous ictal events. Whether HCAR1 influences epileptogenesis leading to chronic recurrent epileptic events has still to be determined. Epileptogenesis is a complex process driven by oxidative stress and neuroinflammation^68^ and involving cellular and network reorganization, that includes ryanodine receptors and various synaptic proteins.^69^ The understanding of factors favoring epileptogenesis will likely benefit from omics approaches, such as the analysis of plasma proteins used as biomarkers in individuals experiencing recent and recurrent seizures.^70^

In this study, we provided extensive evidence showing that lactate and HCAR1 are important players in induced seizure modulation. Together, they exert a tonic inhibitory influence across the brain, most likely by acting on various cell types to mitigate seizure severity. Contrary to prior assumptions, lactate during seizures may not be detrimental but rather mobilized by the brain to leverage HCAR1 action to keep activity under control.

### Limitations of the study

The administration of a synthetic HCAR1 agonist failed to modulate seizures in an *in vivo* epilepsy model, possibly due to competition with elevated endogenous lactate levels during seizures, underscoring the intricate interplay between exogenous and endogenous HCAR1 modulators. The use of acute seizure models in mice may not fully represent the complexity of human epileptic conditions, requiring cautious extrapolation of the findings to clinical settings. Additionally, although drastic differences in recovery time between wild-type and HCAR1-KO mice were observed, the underlying mechanisms of this prolonged recovery in HCAR1-KO mice will require further investigation.

## STAR★Methods

### Key resources table

**Table:** 

REAGENT or RESOURCE	SOURCE	IDENTIFIER
**Chemicals, peptides, and recombinant proteins**		

Sodium chloride (NaCl)	VWR International	Cat.# 27800.291
Potassium chloride (KCl)	Sigma Aldrich	Cat.# P9333-500G
Sodium phosphate monobasic (NaH_2_PO_4_)	Sigma Aldrich	Cat.# 71496-250G
Magnesium sulfate heptahydrate (MgSO_4_)	Sigma Aldrich	Cat.# M5921-500G
Sodium bicabonate (NaHCO_3_)	Sigma Aldrich	Cat.# S5761-1KG
Calcium choride dihydrate (CaCl_2_)	Sigma Aldrich	Cat.# 21097-250G
Glucose	Sigma Aldrich	Cat.# RDD016-1KG
Sodium oxamate	Sigma Aldrich	Cat.# O2751-10G
NHI-2	Sigma Aldrich	Cat.# SML1463
4-Aminopyridine	Tocris	Cat.# 0940
Kainate	Tocris	Cat.# 0222

**Experimental models: Organisms/strains**		

C57BL/6NRj	Janvier Labs	N/A

**Deposited data**		

Original data	Zenodo	https://zenodo.org/records/10838588

**Software and algorithms**		

GraphPad Prism10	Dotmatics	RRID:SCR_002798; https://www.graphpad.com/
MATLAB	Mathworks	RRID:SCR_001622; https://www.mathworks.com/
pCLAMP 10	Molecular Device	RRID:SCR_011323; https://www.moleculardevices.com/
Intan RHX software	Intan technology	https://intantech.com/RHX_software.html
Symply2Read	Matlab script	https://github.com/luthilab/IntanLuthiLab

**Other**		

Stereotaxic apparatus	Kopf Instruments	N/A
DC temperature controller	FHC	Cat.# 40-90-8D
Heating Pad	FHC	Cat.# 40-90-2-02
Kwik-cast	World Precision Instrument	Cat.# KWIK-CAST
Tungsten electrodes	FHC	Cat.# UEWSCGSEBNNE
EEG electrode array	Neuronexus	Cat.# H32
33G Hamilton Neuros syringe	Hamilton	Cat.# 65460-06
32G Hamilton Neuros syringe	Hamilton	Cat.# 65457-02
Intan RHD-32 channel headstage	Intan technology	Cat.# C3324
Intan SPI cable	Intan technology	Cat.# C3211
Intan RHD USB interface board	Intan technology	Cat.# C3100
Surveillance IP cameras	Arecont Vision	Cat.# AV2115DNv1

### Resource availability

#### Lead contact

Further information and requests for resources and reagents should be directed to and will be fulfilled by the Lead Contact, Jean-Yves Chatton (jean-yves.chatton@unil.ch).

#### Materials availability

The study did not generate new reagents.

#### Data and code availability

• Data reported in this paper have been deposited in Zenodo: https://zenodo.org/records/10838588.
• This paper does not report original code.
• Any additional information required to reproduce the data reported in this paper is available from the lead contact upon request.

### Experimental model and study participant details

#### Ethical statement for animal experiments

All animal experimentation procedures were carried out in compliance with the recommendations of the Swiss National Institutional Guidelines on animal experimentation and were approved by the canton of Vaud Cantonal Veterinary Office Committee for Animal Experimentation (Switzerland; Licenses VD1288x7d, VD1288x8d, VD3361c and VD3361x1a) and conformed to the ARRIVE guidelines.

#### Experimental animals

Animals were housed in our local animal facility with *ad libitum* access to food and water (maximum 5 animals per cage) before use for experiments. Due to female resistance to kainate induced seizures, male C57BL/6N (Janvier Lab) and male HCAR1-KO mice (3-4 weeks for acute slice and 7-10 weeks for *in vivo* experiments) were used for experiments. HCAR1-KO mice were generously obtained from Prof. Stefan Offermanns at the Max Planck Institute for Heart and Lung Research, Bad Nauheim, Germany.

### Method details

#### *Ex vivo* electrophysiology

##### Slice preparation

Mice were deeply anesthetized by isoflurane inhalation and promptly decapitated after the disappearance of reflexes. Their brains were quickly extracted and immersed in ice-cold cutting artificial cerebrospinal fluid (aCSF) composed of (mM): 80 NaCl, 2.5 KCl, 1.25 NaH_2_PO_4_, 4.5 MgSO_4_, 25 NaHCO_3_, 0.5 CaCl_2_, 10 glucose, and 90 sucrose. Slices were continuously oxygenated with 95% O_2_ and 5% CO_2_ throughout the entire procedure. Horizontal brain slices, each 300 μm thick, were cut using a vibratome (Leica VT1000s). Subsequently, slices were transferred to the cutting aCSF maintained at room temperature, after an initial recovery period of 20-30 minutes at 34°C in cutting aCSF.

##### Electrophysiological recordings

For electrophysiological recordings, an aCSF solution with the following concentrations (mM) was used: 120 NaCl, 3.2 KCl, 1 NaH_2_PO_4_, 1 MgSO_4_, 26 NaHCO_3_, 2 CaCl_2_, and 10 glucose. Adjustments were then made to this basic solution to accommodate the specific requirements of individual experiments. To induce seizure activity, the slices were subjected to an incubation period ranging from 20 minutes to 1 hour in an aCSF solution devoid of Mg^2+^ and containing 100 μM 4-aminopyridine (4-AP; 0940, Bio-Techne AG, Küng Rechstanwälte, Switzerland) and designated as the “epilepsy” aCSF. In instances where the inhibition of lactate was needed, the epilepsy aCSF was supplemented with 6 μM of the LDHA inhibitor NHI-2 (SML1463, Sigma-Aldrich, Saint-Louis, MO, USA). The concentration of the HCAR1 agonist 3Cl-HBA (#16795, Cayman Chemical, Ann Arbor, MI, USA) was set at 80 μM. After the incubation period, slices were placed in a recording chamber under a Zeiss LSM510 Meta upright microscope equipped with infrared differential interference contrast and a Zeiss 40X water immersion objective, and continuously perfused with the corresponding aCSF at a rate of 6 ml/min. For recording local field potentials (LFP), a borosilicate glass pipette with a resistance of 1-2 MΩ and filled with recording aCSF was placed in the CA3 pyramidal layer. The LFP recordings were conducted in current-clamp configuration using a Multiclamp 700B amplifier (Molecular Devices, San José, CA, USA). Data acquisition was carried out at a sampling rate of 10 kHz, with a 2 kHz low-passe filter applied and controlled with pCLAMP 10 software (RRID:SCR_011323) connected to a Digidata 1440A (Molecular Devices).

#### Stereotaxic surgeries and implantation of electrodes

##### EEG

Mice (7-10 weeks old) underwent stereotaxic surgery for the implantation of EEG electrodes and the creation of craniotomies. Mice were initially anesthetized using 5% isoflurane and then immediately secured in a rodent stereotaxic apparatus (Stoelting, Wood Dale, IL, USA). Body temperature was maintained by a heating pad placed underneath the animal and plugged into a DC temperature controller (FHC, Bowdoin, ME, USA). In an effort to prevent drying of the eyes, a lubricating gel (Viscotears, Bausch & Lomb Swiss AG) was applied to the eyes. A maintenance dose of 2-1.5% isoflurane was administered via a nose cone integrated into the stereotaxic frame for the duration of the surgery. Before any surgical interventions, Buprenorphine (0.1 mg/kg, s.c.) was administered. Bupivacaine (2.5 mg/kg s.c.) and lidocaine (6 mg/kg s.c.) were injected at incision sites to manage pain. The scalp was then swabbed with 70% ethanol and shaved using shaving cream.

Following a midline scalp incision, two craniotomies were drilled above the hippocampi at -1.9 mm anterior-posterior and ±1.5 mm medial-lateral relative to bregma. The craniotomies were then sealed with a low-viscosity silicon sealant (Kwik-cast, WPI, Sarasota, FL, USA). An additional pair of craniotomies were drilled posterior to the initial ones to accommodate subdural gold-plated EEG electrodes, made from 0.06 mm copper wire and gold plated by immersion in a potassium gold cyanide solution overnight. Additionally, two reference silver wires were positioned above the cerebellum. Secure fixation of the electrodes was achieved with the application of cyanoacrylate glue. A generic 10-pin connector was centrally placed atop the skull, with the electrodes securely embedded in dental cement (Palavit G). A recovery period of at least one week was granted to the mice before any experimental procedures were initiated.

##### Local field potential

During the same age range of 7 to 10 weeks, mice underwent stereotaxic surgery for LFP electrode implantation. Employing a procedure similar to that outlined above, a midline scalp incision was performed, and two craniotomies were executed at the coordinates of -2.05 mm anterior-posterior and ±2 mm medial-lateral. Subsequently, two 76 μm thick tungsten electrodes with 300-500 kΩ impedance (UEWSCGSEBNNE, FHC, Bowdoin, ME, USA) were introduced to the CA3 pyramidal layer at a depth of -2.25 mm dorsal-ventral. Two reference silver wires were placed above the cerebellum. The electrodes were secured to the skull using cyanoacrylate glue, after which they were soldered to a 10-pin connector and embedded within dental cement. Similar to the EEG protocol, the mice were allowed a recovery period of at least one week before any experimental activities.

##### EEG electrode array

Between the ages of 7 to 10 weeks, mice were subjected to stereotaxic surgery for the implantation of EEG electrode array, following the same procedure established by Jonak et al.^71^ Briefly, a midline scalp incision was performed to expose the skull. A 0.3% hydrogen peroxide solution was applied to clean the surface of the skull. Two holes were drilled on the occipital bone and one hole was drilled above the olfactory bulbs. Three golden screws were then placed in these holes. The left dorsal screw was used as ground and reference. A polyimide 30 channels EEG electrode array (H32, Neuronexus, Ann Arbor, MI, USA) was placed on the surface of the skull. Subsequently, a Teflon sheet was placed atop the grid, extending through the screws to serve as a protective layer. The application of dental cement to confer structural integrity to the implant was then performed. Atop this construct, the omnetics-style connector was positioned and fixed to two wooden sticks for stability using cyanoacrylate glue. Mice were given at least 1 week of recovery before any experiment.

#### Seizure induction

The selection of the kainate application methods was guided by a combination of scientific, practical, and ethical considerations, with each model offering unique advantages in addressing different aspects of our research objectives while prioritizing animal welfare and experimental consistency.

##### HCAR1 agonist

In light of the limited bioavailability and blood-brain barrier permeability data available on HCAR1 agonists, we opted for direct hippocampal injection. At least one-week post-implantation, mice were placed in observatory cages and plugged to the EEG recording system one day prior to the seizure induction process to collect a 24h baseline recording. On the designated day for seizure induction, the mice were placed on a stereotaxic frame under isoflurane anesthesia. After the removal of the silicone covering the craniotomies, a Hamilton Neuros syringe with a 33-gauge needle (65460-06, Hamilton, Bonaduz, Switzerland) was carefully lowered to target the dentate gyrus within both hippocampi, at a depth of -2.0 mm dorsal-ventral. 500 nl of saline solution containing 2.5 mM of 3Cl-HBA, was injected at a controlled rate of 100 nl/min. To avoid reflux during the withdrawal of the syringe, it was maintained *in situ* for an additional 5 minutes. Concluding this step, a 32-gauge Hamilton Neuros syringe (65457-02, Hamilton, Bonaduz, Switzerland) with a volume of 0.5 μl was lowered to reach the CA1 pyramidal layer within the right dorsal hippocampus, at a depth of -1.0 mm dorsal-ventral. In this case, a solution consisting of 20 mM of kainate (#0222, Bio-Techne AG, Switzerland) and 2.5 mM of 3Cl-HBA in saline, totaling 50 nl, was injected at a controlled rate of 50 nl/min. The syringe remained in place for an additional 5 minutes before withdrawal. The mouse was then plugged into the EEG recording system during the recovery from anesthesia.

##### Seizure threshold determination

For the determination of seizure threshold, mice were placed in observatory cages on the day of the experiment and were allowed one hour of undisturbed acclimatization to their new environment. Subsequently, injections of saline solution containing kainate at 5 mg/kg intraperitoneally were administered at 30-minute intervals. The resultant behavioral seizures were evaluated in accordance with a modified Racine scale, with scores ranging from 1 (behavioral arrest) to 6 (clonic, tonic-clonic seizure with wild jumping). The quantification of the time and dosage required to instigate a seizure rated at 4 (Bilateral forelimb clonic seizure with rearing/sitting) on the Racine scale was quantified.

##### Acute seizures

After an interval of at least one week following LFP and EEG probe implantation surgery, mice were introduced into observatory cages and linked to the EEG recording system one day prior to initiating acute seizures. Baseline EEG recordings spanning 24 hours were obtained. To induce acute seizures, two subcutaneous kainate injections were administered, spaced an hour apart. The observed susceptibility differences in kainate-induced seizures led to the implementation of dual kainate injections at 10 mg/kg in knockout (KO) mice and at 15 mg/kg in the wild-type (WT) counterparts. A subset of WT and KO mice received an identical dose (10mg/kg) to highlight the high seizure susceptibility of KO mice and adequately compare seizure number and duration, and their EEG response pattern. Recordings were performed for 3 hours (or until the death of the animal) and were terminated if the animal did not experienced seizures for an entire hour.

#### EEG recordings

A minimum recovery period of 1 to 2 weeks was allocated to all mice before EEG recordings. During this recovery interval, each mouse was accommodated within a standard housing cage at our animal facility with unrestricted access to food and water. The day preceding the recording session, mice were transferred into cylindrical Plexiglas cages, (25 cm diameter 20.5 cm height) placed inside a Faraday cage. In preparation for the recordings, a pre-amplifier (Intan RHD-32 channel headstage, model C3324, Intan Technologies, Los Angeles, CA, USA) was connected to the 10-pin headcap or the omnetic connector present on the EEG probe. After this, the headstage was linked to an electrical slip ring through an Intan SPI cable (model C3211), then connected to an Intan RHD USB interface board (model C3100). The EEG signals were captured via a custom MATLAB-based acquisition system (Symply2Read, available at https://github.com/luthilab/IntanLuthiLab, MathWorks, Natick, MA, USA) at a sampling rate of 1000 Hz. Some recordings were acquired through Intan RHX software (accessible at https://github.com/Intan-Technologies/Intan-RHX) at a rate of 2000 Hz. To mitigate power line interference, the EEG signals were subjected to notch filtering at 50 Hz during the acquisition process and a Faraday cage was built around the system. Video recordings were simultaneously executed via surveillance IP cameras (model AV2115DNv1, Arecont Vision, Clovis, CA, USA). Recordings were conducted for at least 3 hours or until the sudden death of the animal during a seizure. Recordings were terminated if the animal did not experience a seizure for an hour. Seizures were automatically detected by offline analysis.

### Quantification and statistical analysis

#### EEG quantification

All EEGs were analyzed offline with custom MATLAB scripts. Seizures were defined as rhythmic (> 3Hz) oscillations that lasted > 5s and were at least 3 standard deviations above the baseline root mean square (RMS) amplitude.^11^ For LFP, the channel displaying the higher number of seizures was used for power analysis. Each seizure was considered as a single trial and each trial was synchronized to seizure onset. The window of analysis was -5 seconds to 40 seconds in the case of LFP recording and -5 seconds to 30 seconds for the 30 channels EEG probe.

##### Power spectrum analysis

Quantification of EEG power was executed offline through the application of complex Morlet wavelet convolution (for review see: Cohen, 2019).^72^ In short, a series of complex Morlet wavelets were generated as follows:

$$\omega =e^{i2\pi ft}e^{-\frac{t^{2}}{2\sigma^{2}}}$$

Where:

$$\sigma =\frac{n}{2\pi f}$$

Where n denotes the number of cycles ranging from 1 to 30 in increments of 200 steps. The power spectrum of both the signal and the wavelet was obtained via the fast Fourier transform (FFT) algorithm. Subsequently, convolution was carried out in the frequency domain through element-wise multiplication of the Morlet wavelet and the signal spectrums. The power was then extracted as the magnitude of the resulting Fourier coefficients. The frequency bands common to EEG studies of this kind were kept for further analysis: delta (1-3Hz), theta (4-8Hz), alpha (9-13Hz), beta (14-30Hz) gamma (31-80Hz), and high-frequency oscillations (HFO, 81-200Hz).

Detected seizures were aligned according to their onset. A five second time window was taken before seizure onset and a 30 (EEG electrode array) or 40 seconds (LFP recording) time window was taken after seizure onset. For the LFP recordings, each seizure was considered as a trial whereas in the case of EEG electrode array recordings, the individual seizures had to be average to maintain data size within a manageable range.

For statistical analysis, GraphPad Prism 10 (GraphPad Software, Boston, MA, USA) was used. All the statistical details of experiments can be found in the corresponding figure legend, including the statistical tests used, exact value of n, and what n represents. Values are given as means ± SEM and the statistical significance threshold was set as p<0.005.
