# Supplementary material for: The lactate receptor HCAR1: A key modulator of epileptic seizure activity
Source: iScience. 2024 Apr 6;27(5):109679. doi: 10.1016/j.isci.2024.109679 (PMC11035371; doi:10.1016/j.isci.2024.109679)
Supplement: Document S1. Figures S1–S3 [file mmc1.pdf]

iScience, Volume *27*

Supplemental information

The lactate receptor HCAR1: A key modulator of epileptic seizure activity

Maxime Alessandri, Alejandro Osorio-Forero, Anita Lüthi, and Jean-Yves Chatton

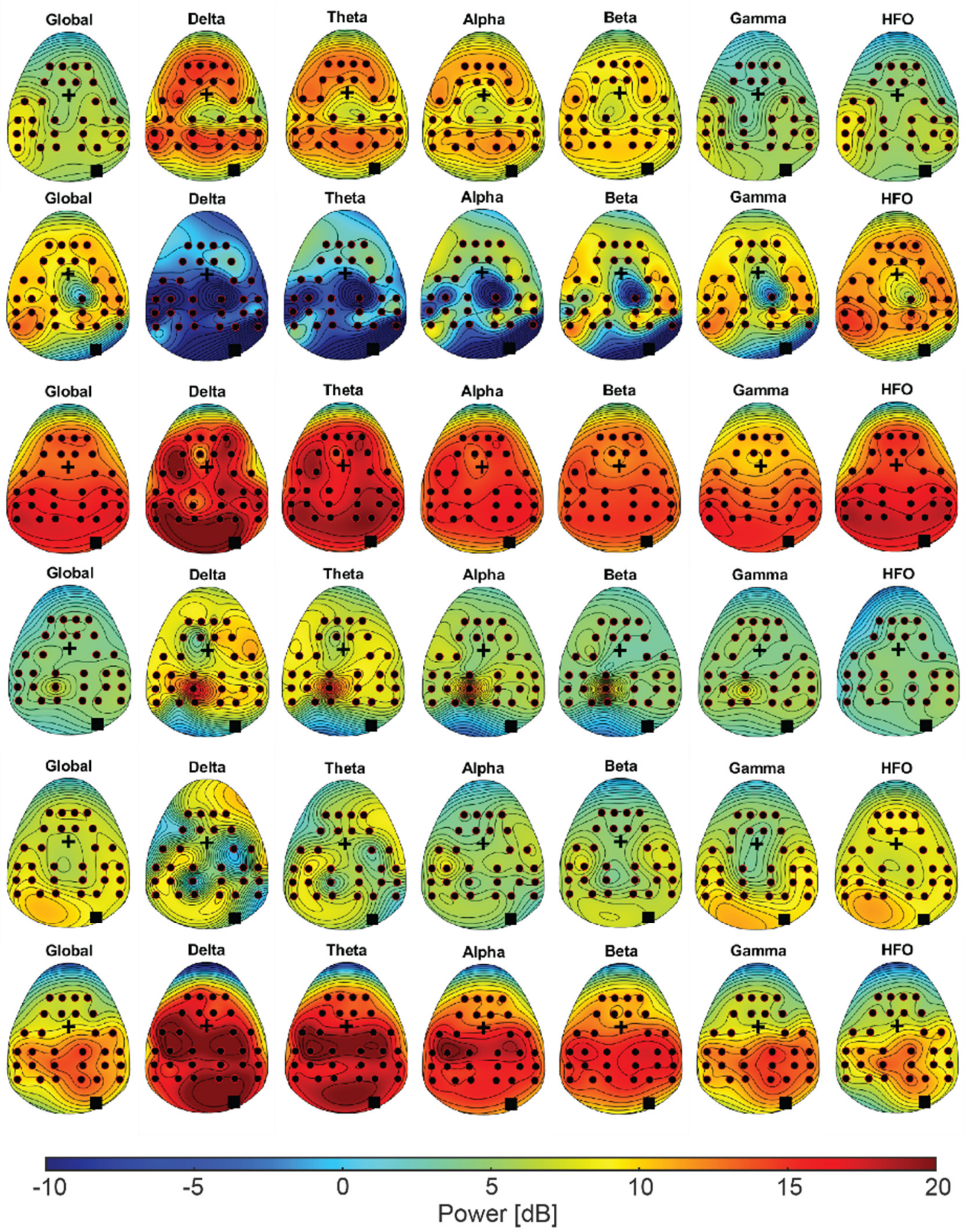

Figure S1. Spatial seizure power spread for individual WT mice, related to Figure 4.

Topographic map representing seizure power in WT mice. Each row represents one mouse.

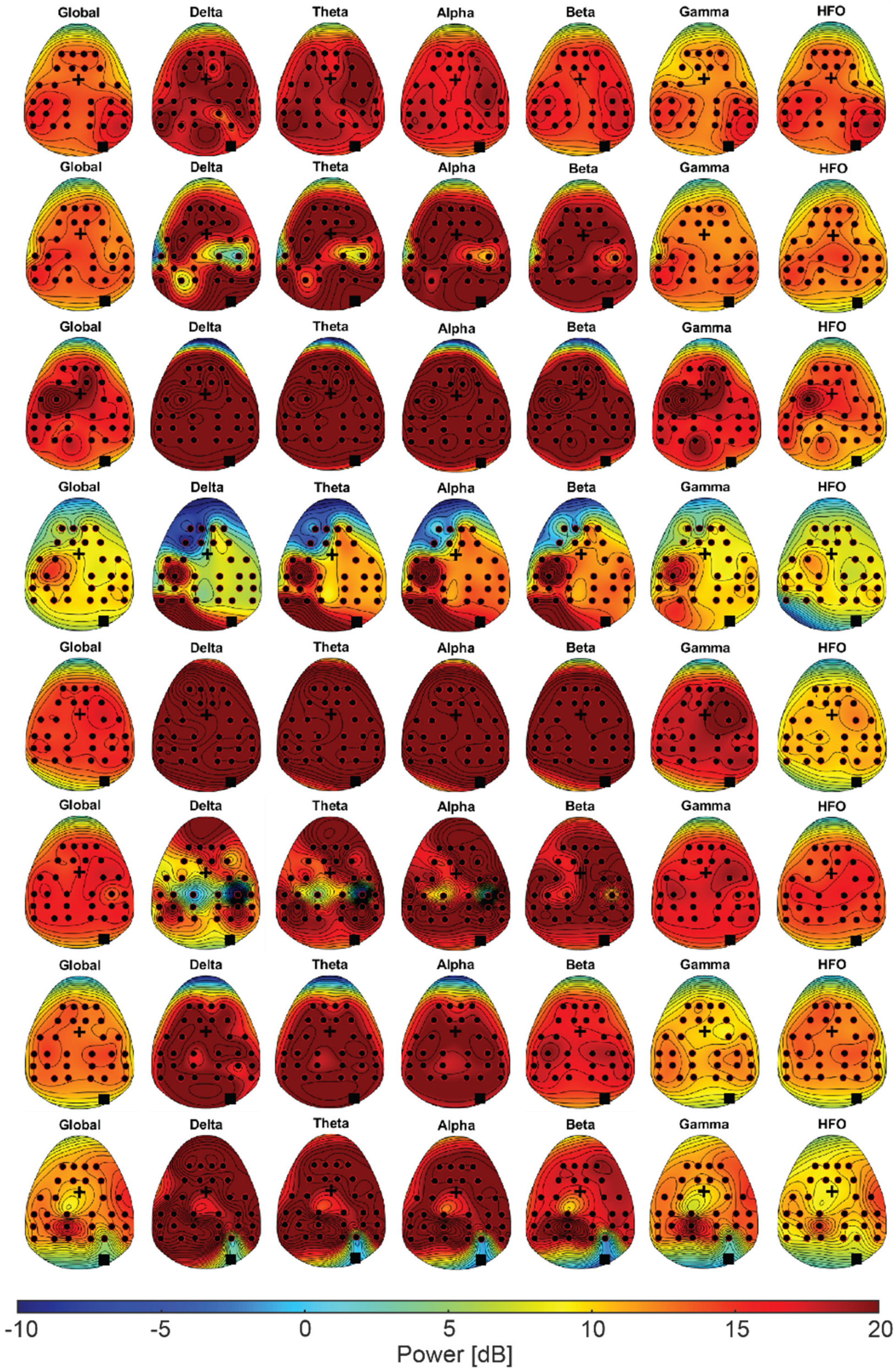

Figure S2. Spatial seizure power spread for individual HCAR1-KO mice, related to Figure 4.

Topographic map representing seizure power in WT mice. Each row represents one mouse.

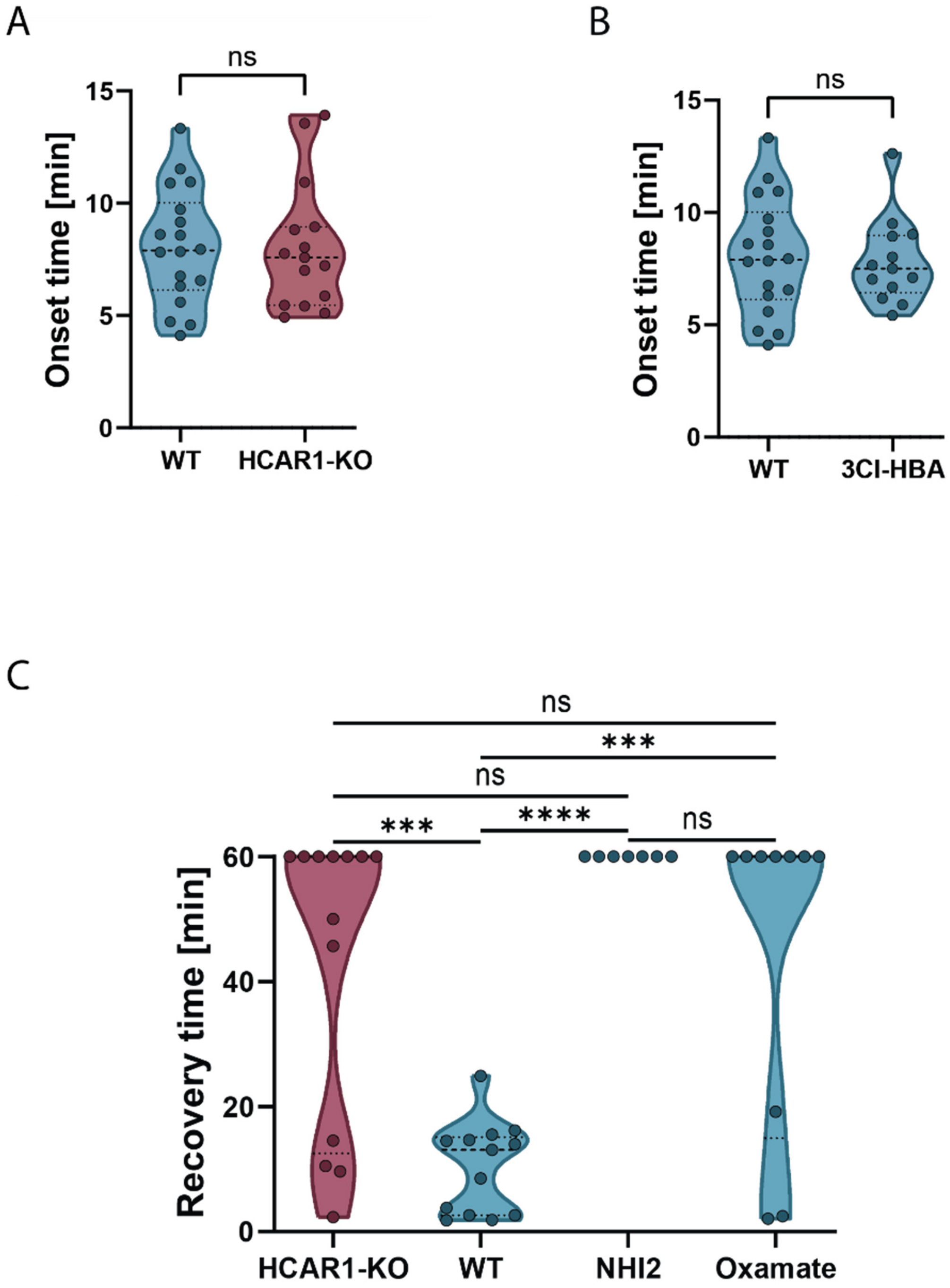

Figure S3. Seizure onset time *ex vivo and LDH inhibition*, related to Figure 6.

(A-B) Time required for the first epileptic-like event to occur after perfusion of epileptic aCSF, i.e., seizure onset. **(A)** Seizure onset time in WT vs HCAR1-KO mice. Means ± SEM: WT = 8 ±0.6, HCAR1-KO = 8±0.7 $p > 0.005$ n = 18 WT, n = 13 WT + 3Cl-HBA, n = 15 KO. **(B)** Seizure onset time in WT in the presence and absence of HCAR1 agonist. Means ± SEM: WT = 8 ±0.6, 3Cl-HBA (80µm) = 7.8±0.5, n = 18 WT, n = 13 3Cl-HBA **(C)** Recovery time in HCAR1-KO mice and WT when perfused with the selective inhibitor of LDHA, NHI-2 (6µM) or the ubiquitous LDH inhibitor oxamate (10mM). Means ± SEM: HCAR1-KO = 8±0.7 n = 13, WT = 8 ±0.6 n = 13, NHI-2 = 60.00 n = 7, oxamate = 44.37±8 n = 10. $p = 0.004$ HCAR1-KO vs. WT, $p < 0.0001$ WT vs. NHI2, $p = 0.0005$ WT vs. oxamate.
